# Preoperative Esophageal Stenting and 5-Year Survival in Patients Undergoing Esophagectomy for Esophageal Cancer: a Population-Based Nationwide Study from Finland

**DOI:** 10.1007/s11605-023-05643-7

**Published:** 2023-03-07

**Authors:** Olli Helminen, Eero Sihvo, Mika Helmiö, Heikki Huhta, Raija Kallio, Vesa Koivukangas, Arto Kokkola, Simo Laine, Elina Lietzen, Sanna Meriläinen, Vesa-Matti Pohjanen, Tuomo Rantanen, Ari Ristimäki, Jari V. Räsänen, Juha Saarnio, Vesa Toikkanen, Tuula Tyrväinen, Antti Valtola, Joonas H. Kauppila, on behalf of the FINEGO group

**Affiliations:** 1 Surgery Research Unit, Medical Research Center Oulu, Oulu University Hospital, University of Oulu, Oulu, Finland; 2 Department of Surgery, Oulu University Hospital, Kajaaninkatu 50, 90220 Oulu, Finland; 3 Department of Surgery, Central Finland Central Hospital, Jyväskylä, Finland; 4 Division of Digestive Surgery and Urology, Turku University Hospital, Turku, Finland; 5 Department of Oncology and Radiotherapy, Oulu University Hospital, Oulu, Finland; 6 Department of Surgery, Helsinki University Hospital, University of Helsinki, Helsinki, Finland; 7 Cancer and Translational Medicine Research Unit, Medical Research Center Oulu, Oulu University Hospital, University of Oulu, Oulu, Finland; 8 Department of Surgery, Kuopio University Hospital, University of Eastern Finland, Kuopio, Finland; 9 Department of Pathology and HUSLAB, Helsinki University Hospital, University of Helsinki, Helsinki, Finland; 10 Applied Tumor Genomics Research Program, Research Programs Unit, University of Helsinki, Helsinki, Finland; 11 Department of General Thoracic and Oesophageal Surgery, Heart and Lung Centre, Helsinki University Hospital, University of Helsinki, Helsinki, Finland; 12 Department of Cardiothoracic Surgery, Heart Center, Tampere University Hospital, University of Tampere, Tampere, Finland; 13 Department of Gastroenterology and Alimentary Tract Surgery, Tampere University Hospital, Tampere, Finland; 14 Upper Gastrointestinal Surgery, Department of Molecular Medicine and Surgery, Karolinska Institutet, Karolinska University Hospital, Stockholm, Sweden

**Keywords:** Esophageal stent, Esophageal cancer, Bridge to surgery, Survival, Esophagectomy, Nutrition

## Abstract

**Background:**

Preoperative esophageal stenting is proposed to have a negative effect on outcomes. The aim was to compare a 5-year survival in patients undergoing esophagectomy for esophageal cancer with and without preoperative esophageal stent in a population-based nationwide cohort from Finland. The secondary outcome was 90-day mortality.

**Methods:**

This study included curatively intended esophagectomies for esophageal cancer in Finland between 1999 and 2016, with follow-up until December 31, 2019. Cox proportional hazards models provided hazard ratios (HRs) with 95% confidence intervals (CIs) of overall 5-year and 90-day mortality. Model 1 was adjusted for age, sex, year of the surgery, comorbidities, histology, pathological stage, and neoadjuvant therapy. Model 2 included also albumin level and BMI.

**Result:**

Of 1064 patients, a total of 134 patients underwent preoperative stenting and 930 did not. In both adjusted models 1 and 2, higher 5-year mortality was seen in patients with preoperative stent with HRs of 1.29 (95% CI 1.00–1.65) and 1.25 (95% CI 0.97–1.62), respectively, compared to no stenting. The adjusted HR of 90-day mortality was 2.49 (95% CI 1.27–4.87) in model 1 and 2.49 (95% CI 1.25–4.99) in model 2. When including only neoadjuvant-treated patients, those with preoperative stent had a 5-year survival of 39.2% compared to 46.4% without stent (adjusted HR 1.34, 95% CI 1.00–1.80), and a 90-day mortality rate of 8.5% and 2.5% (adjusted HR 3.99, 95% CI 1.51–10.50).

**Discussion:**

This nationwide study reports worse 5-year and 90-day outcomes in patients with preoperative esophageal stent. Since residual confounding remains possible, observed difference could be only an association rather than the cause.

## Introduction

Worldwide, esophageal cancer is the sixth leading cause of cancer-related death.^1^ In local and locally advanced disease, curative treatment is possible with surgery.^2^ Before surgery, dysphagia is commonly present, especially in large and obstructing tumors, leading to weight loss and malnutrition.^3^

Malnutrition is associated with postoperative complications, reoperations, and decreased long-term survival.^4,5^ To improve survival, neoadjuvant therapy is recommended for all locally advanced diseases, i.e., T3 and/or N + esophageal cancers,^2,6^ and stenting, during this period, is able to secure enteral nutrition.^7,8^ Alternatives for stenting include nasogastric tube, feeding jejunostomy, and gastrostomy.^9–11^ Stenting is commonly used due to its simplicity. Stent can, however, cause adverse events,^12,13^ and preoperatively placed stent is proposed to increase short-term complications^2,14^ and 90-day mortality based on registry data.^15^ In a matched cohort study with 38 stented patients from high-volume European centers, stented patients had more serious complications (Clavien-Dindo ≥ 3a) and increased risk estimates for in-hospital mortality, although not statistically significant.^14^ In that matched cohort study, an overall 3-year survival was reduced in stented patients.^14^ There is a need for larger studies assessing the short- and long-term outcomes associated with preoperative esophageal stenting in patients who undergo surgery for esophageal cancer.

The primary aim of this study was to compare the 5-year survival in patients with and without preoperative esophageal stent adjusted for confounding factors in a population-based cohort from Finland. The secondary outcome was 90-day mortality.

## Materials and Methods

### Study Design

This was a population-based, nationwide, and retrospective cohort study from Finland including esophagectomy for esophageal adenocarcinoma or squamous cell carcinoma. The study period was from January 1999 to December 2016, with follow-up until December 31, 2019.^16^ Patients with preoperative esophageal stent were compared to those without preoperative stent in relation to an overall 5-year survival as the primary outcome. The study was approved by the Regional Ethical Review Board in Oulu, Finland, the Finnish national health officials, and hospital districts.^16^

### Data Collection

The Finnish National Esophago-Gastric Cancer Cohort (FINEGO) includes all patients with esophageal and gastric cancer diagnosed in Finland between 1987 and 2016, identified from the Finnish Cancer Registry and Hospital Discharge Registry,^16^ which are 92% and 98% complete for esophageal cancer, respectively.^17^ The identification using both registries by searching for cancer diagnoses and operation codes allows 100% completeness on patient identification. To Finnish Cancer Registry, a new cancer diagnosis is automatically reported from pathological laboratories. This and compulsory reporting of performed procedures with the diagnosis by the hospitals, linked to hospital funding, to Hospital Discharge Registry makes patient identification reliable.^17^ Furthermore, instead of having a solely registry-based study, we were able to retrieve and review all patient medical records, making this nationwide data unique compared to that of previous registry-based studies. Of patient records, 9% were missing and this is a possible source of bias. These missing records are, however, unlikely to cause selection bias since the use of preoperative stent should not be linked to missing patient records. After identification of cases, available information including age, sex, comorbidity,^18^ surgery, and other variables was collected from the Finnish Cancer Registry, Finnish National Institute for Health and Welfare registries, Care Register for Healthcare, and Hospital Discharge Registry.^16^ Medical reports were obtained from the respective healthcare units and reviewed by specialized surgeons, providing accurate information on the type of resection, tumor location, histology, stage and size, neoadjuvant treatment, laboratory values including albumin and prealbumin, and the use of preoperative esophageal stents. All-cause mortality data was obtained from the 100% complete death registry, held by Statistics Finland until December 31, 2019.^16^

### Exposures

The study exposure was preoperative stent (exposure group), which was compared to patients with no-stent (control group).

### Outcomes

The primary outcome of the study was overall 5-year survival. The secondary outcome was 90-day mortality.

### Statistical Analysis

The analyses followed a detailed a priori study protocol. IBM SPSS v26.0 (IBM Corp., Armonk, NY) was used for all analyses. Follow-up times were calculated from the date of surgery until the time of death or the end of follow-up, whichever occurred first. Survival was calculated using the life table method, visualized with Kaplan–Meier curves. Cox proportional hazards models provided hazard ratios (HRs) with 95% confidence intervals (CIs). To avoid confounding, two models of adjustments for seven known prognostic factors were made: age (continuous), sex (male/female), year of surgery (continuous), comorbidity (Charlson Comorbidity Index^18^ 0, 1, or ≥ 2 (excluding esophageal cancer under treatment)), histological type of cancer (adenocarcinoma or squamous cell carcinoma), neoadjuvant therapy (yes/no), and pathological stage (stages 0–I, II, III, and IV, according to the 8th edition AJCC/UICC staging of cancers of the esophagus and esophagogastric junction^19^). Model 2 aimed to control confounding related to malnutrition, including albumin and BMI. Abnormal albumin (yes or no) was defined as < 34 g/l or prealbumin defined as < 0.24 g/l, if information on albumin level was not available. Both low and high BMIs as risk factors for postoperative morbidity and mortality have been previously reported in a nationwide study.^20^ In our study with western population, high-risk BMI (yes or no) was defined as < 18.5 kg/m^2^ or ≥ 35 kg/m^2^ according to WHO criteria for underweight and severe obesity. Furthermore, the following subgroup analyses were performed: (1) locally advanced disease (cT3 and/or cN1) patients to include only candidates for neoadjuvant treatment and worse prognosis, (2) patients who received neoadjuvant therapy to homogenize comparison related to the extent of the disease and also physical fitness, (3) cT3 and cT4 tumors, and (4) only large tumors (≥ 50 mm). The adjustments for the subgroups were performed as described above. Tumor size was acquired from the final pathology report in patients not receiving neoadjuvant therapy. In patients who received neoadjuvant, imaging and endoscopy reports were used in tumor size determination.

Patients with completely missing medical records or unclear exposure information were excluded from the main analysis. Missing confounder data were handled by conducting both complete case analysis and multiple imputation. For multiple imputations, the number of imputations was 20. Imputed variables included histology (4 imputed values), pathological stage (18 imputed values), pT stage (14 imputed values), pN stage (7 imputed values), neoadjuvant treatment (6 imputed values), tumor size (110 imputed values), BMI (164 imputed values), and albumin (567 imputed values). Previously, multiple imputation has been demonstrated effective even in the case of a high proportion of missing values,^21^ such as albumin in this study. There were no differences in results of complete case analysis and multiple imputation, and therefore, only the imputed results are presented.

## Results

### Patients

A total of 1235 patients who underwent esophagectomy from 1999 to 2016 with the diagnosis of esophageal cancer were identified. Of these, 1124 patient records were available for the analysis. The cause for exclusion was non-squamous cell or non-adenocarcinoma histology in 38 patients, non-primary tumor or non-resected disease in 7, and gastrectomy without esophagectomy in 14. One patient lacked the information about stenting. Therefore, the final study group included 1064 patients who underwent esophagectomy and had preoperatively placed stent (*n* = 134) or not (*n* = 930). Indication for stenting was severe dysphagia with inability to eat solid food or liquids in all cases. Other invasive feeding routes were seldom used (2 patients had preoperative feeding jejunostomy and none percutaneous gastrostomy).

Baseline characteristics are provided in Table 1. Patients with esophageal stent were slightly older (median 66 years) than no-stent group (64 years) and had lower BMI (median 21.8 compared to 25.8) and more often abnormal albumin levels (64.5% compared to 17.5%). In patients with preoperative stent, margin positive (R1/2) resection rate was 13.4% compared to 10.9% without stent. Operation time was longer in the stent group with a median of 349 min compared to 319 min. No major differences were seen in bleeding, hospital stay, or ICU stay (Table 1).

**Table 1:** Characteristics of 1064 patients having undergone surgery for esophageal cancer with and without preoperative stent in Finland in 1999–2016

Variable	All operations	No preoperative stent	With preoperative stent
	No. of patients (%) *n* = 1064	No. of patients (%) *n* = 930	No. of patients (%) *n* = 134
Year of the operation			
1999–2001	128 (12.0)	125 (13.4)	3 (2.2)
2002–2004	137 (12.9)	126 (13.5)	11 (8.2)
2005–2007	144 (13.5)	132 (14.2)	12 (9.0)
2008–2010	183 (17.2)	163 (17.5)	20 (14.9)
2011–2013	214 (20.1)	178 (19.1)	36 (26.9)
2014–2016	258 (24.2)	206 (22.2)	52 (38.8)
Age, median (IQR)	64 (58–71)	64 (58–71)	66 (59–70)
BMI, median (IQR)	25.4 (22.2–28.4)	25.8 (22.9–28.7)	21.8 (20.0–25.5)
BMI < 18.5 or > 35	77 (8.6)	59 (7.6)	18 (15.0)
Sex			
Male	791 (74.3)	686 (73.8)	105 (78.4)
Female	273 (25.7)	244 (26.2)	29 (21.6)
Charlson comorbidity score			
0	610 (57.3)	536 (57.6)	74 (55.2)
1	299 (28.1)	260 (28.0)	39 (29.1)
≥ 2	155 (14.6)	134 (14.4)	21 (15.7)
Tumor histology			
Adenocarcinoma	750 (70.8)	662 (71.4)	88 (66.2)
Squamous cell carcinoma	310 (29.2)	265 (28.6)	45 (33.8)
Anastomosis location			
Intrathoracic	677 (63.6)	584 (62.8)	93 (69.4)
Neck	379 (35.6)	340 (36.6)	39 (29.1)
Clinical T stage 3 or 4	643 (60.4)		
Clinically locally advanced disease (cT3 and/or N +)	696 (65.4)		
Tumor size, mm, median (IQR)	35 (20–55)	30 (15–50)	50 (30–65)
< 50 mm	557 (58.4)	500 (60.5)	57 (44.9)
> 50 mm	397 (41.6)	327 (39.5)	70 (55.1)
R1/2 resection	119 (11.2)	101 (10.9)	18 (13.4)
Pathological t stage			
0	103 (9.8)	85 (9.2)	18 (13.7)
1	228 (21.7)	221 (24.0)	7 (5.3)
2	226 (21.5)	206 (22.4)	20 (15.3)
3	402 (38.3)	326 (35.5)	76 (58.0)
4	91 (8.7)	81 (8.8)	10 (7.6)
Pathological n stage			
0	622 (58.8)	547 (59.3)	75 (56.0)
1	214 (20.2)	183 (19.8)	31 (23.1)
2	131 (12.4)	112 (12.1)	19 (14.2)
3	90 (8.5)	81 (8.8)	9 (6.7)
Pathological stage			
0–I	335 (32.0)	307 (33.5)	28 (21.7)
II	178 (17.0)	160 (17.4)	18 (14.0)
III	405 (38.7)	338 (36.9)	67 (51.9)
IV	128 (12.2)	112 (12.2)	16 (12.4)
Neoadjuvant treatment			
Yes	502 (47.4)	396 (42.9)	106 (79.1)
No	556 (52.6)	528 (57.1)	28 (20.9)
Preoperative albumin			
Normal	381 (76.7)	359 (82.5)	22 (35.5)
Abnormal	116 (23.3)	76 (17.5)	40 (64.5)
Operative approach			
Open	774 (72.7)	696 (74.8)	78 (58.2)
Hybrid	56 (5.3)	46 (4.9)	10 (7.5)
MIE	234 (22.0)	188 (20.2)	46 (34.3)
Operation time	325 (255–384)	319 (254–380)	349 (280–405)
Bleeding	500 (250–900)	500 (250–900)	500 (300–800)
Hospital stay	15 (12–21)	15 (12–21)	16 (12–22)
ICU stay	2 (1–4)	2 (1–5)	2 (1–4)

Of 134 patients who had preoperative esophageal stent, 106 (79.1%) had neoadjuvant treatment. The reasons for no neoadjuvant treatment despite stenting were as follows: Medical oncologist refused neoadjuvant due to comorbidities (*n* = 10); emergency or urgent surgery due to stent insertion–related tumor perforation (*n* = 9), stent inserted before referral but multidisciplinary team decided to operate without neoadjuvant (*n* = 7), and esophageal stricture treated with multiple dilatations and stenting and eventually esophagectomy which revealed squamous cell cancer (*n* = 1); and early cancer treated with mucosal resection complicated with bleeding and stent was inserted. Pathology revealed T1 cancer with risk factors, and esophagectomy was later performed (*n* = 1).

### Primary Outcomes

**Fig. 1:**
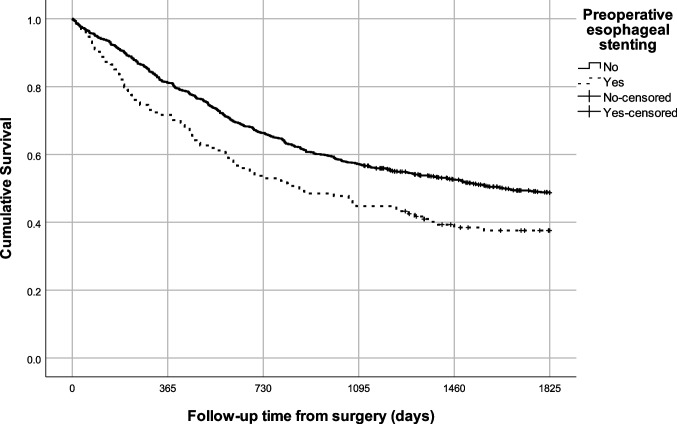
Kaplan–Meier curves of the 5-year survival of patients who underwent esophagectomy for esophageal cancer stratified by preoperative esophageal stent

The observed 5-year survival was 37.6% in patients with preoperative stent and 48.7% in those without stent (*p* = 0.002) (Fig. 1). After adjustment for confounding factors, higher mortality hazard was observed in patients with preoperative stent compared to no stenting in model 1 (HR 1.29, 95% CI 1.00–1.65) and trend for higher mortality hazard in model 2 (HR 1.25, 95% CI 0.97–1.62) (Table 2).

**Table 2:** Hazard ratios (HRs) with 95% confidence intervals (CIs) of 90-day and 5-year mortality comparing patients with esophageal cancer with and without preoperative stent in Finland in 1999–2016

	Number of patients	With preoperative stent, HR (95% CI)	No preoperative stent, HR (95% CI)
Overall 5-year mortality			
All patients (crude)	1064	1.44 (1.14–1.81)	1.00 (reference)
All patients (adjusted)*	1064	1.29 (1.00–1.65)	1.00 (reference)
All patients (adjusted)**	1064	1.25 (0.97–1.62)	1.00 (reference)
Overall 90-day mortality			
All patients (crude)	1064	1.94 (1.05–3.59)	1.00 (reference)
All patients (adjusted)*	1064	2.49 (1.27–4.87)	1.00 (reference)
All patients (adjusted)**	1064	2.49 (1.25–4.99)	1.00 (reference)

^*^Model 1: adjusted for age (continuous), sex, year of the surgery (continuous), Charlson comorbidity score (0, 1, or ≥ 2), histology, pathological stage (0–I, II, III, or IV), and neoadjuvant therapy (yes, or no)

^**^Model 2: adjusted for abovementioned variables, albumin level (normal, abnormal), and BMI (normal, abnormal)

In subgroup analyses, the 5-year survival of locally advanced disease was 38.7% in patients with preoperative stent and 42.4% in patients without stent (*p* = 0.215). In adjusted analysis, those with preoperative stent were associated with higher mortality compared to no stenting (HR 1.34, 95% CI 1.02–1.76) (Table 3).

**Table 3:** Hazard ratios (HRs) with 95% confidence intervals (CIs) of 5-year mortality comparing patients with esophageal cancer with and without preoperative stent in Finland in 1999–2016. Four subgroup analyses were performed

5-year overall mortality	Number of patients	With preoperative stent, HR (95% CI)	No preoperative stent, HR (95% CI)
Locally advanced disease (T3 and/or N +)			
Overall mortality (5 years)			
All patients (crude)	696	1.18 (0.91–1.52)	1.00 (Reference)
All patients (adjusted)*	696	1.34 (1.02–1.76)	1.00 (Reference)
Neoadjuvant-treated patients			
Overall mortality (5 years)			
All patients (crude)	502	1.34 (1.01–1.77)	1.00 (Reference)
All patients (adjusted)*	502	1.34 (1.00–1.80)	1.00 (Reference)
Clinical T3–4 tumors			
Overall mortality (5 years)			
All patients (crude)	643	1.18 (0.91–1.52)	1.00 (Reference)
All patients (adjusted)*	643	1.35 (1.03–1.77)	1.00 (Reference)
Large tumors (≥ 50 mm)			
Overall mortality (5 years)			
All patients (crude)	442	1.13 (0.82–1.56)	1.00 (Reference)
All patients (adjusted)*	442	1.20 (0.86–1.69)	1.00 (Reference)

^*^Adjusted for age (continuous), sex, year of the surgery (continuous), Charlson comorbidity score (0, 1, or ≥ 2), histology, pathological stage (0–I, II, III, or IV), and neoadjuvant therapy (yes, or no). The adjustments included the following: In locally advanced disease, stage was not adjusted for; in neoadjuvant-treated patients, neoadjuvant therapy was not adjusted for; and in clinical T3–4 tumors instead of pathological stage, N stage (0, 1, 2, 3) was adjusted for

**Fig. 2:**
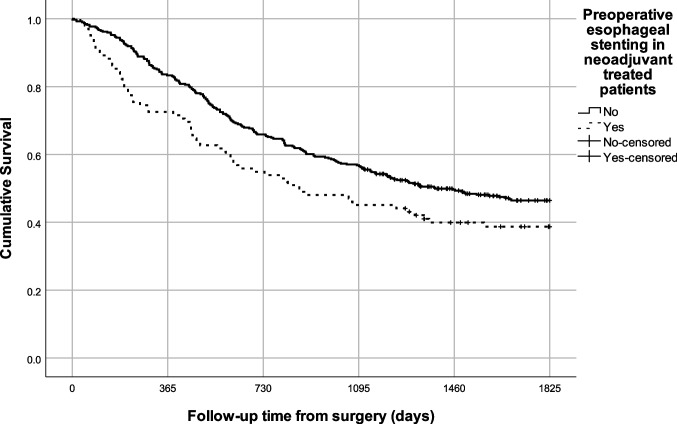
Kaplan–Meier curves of the 5-year survival of patients who underwent esophagectomy for esophageal cancer and received neoadjuvant therapy, stratified by preoperative esophageal stent

When including only neoadjuvant-treated patients, those with preoperative stent had a 5-year survival of 39.2% compared to patients without stent 46.4% (*p* = 0.036) (Fig. 2). In adjusted analysis, higher mortality hazard was seen after preoperative stenting, compared to no stenting (HR 1.34, 95% CI 1.00–1.80) (Table 3).

In the subgroup analysis of patients with clinical T3–4 tumors, the 5-year survival was 37.5% after preoperative stent and 41.5% without stent (*p* = 0.209). In the adjusted analysis, higher mortality hazard was seen after preoperative stent (HR 1.35, 95% CI 1.03–1.77) (Table 3).

In the subgroup analysis of patients with large tumors (≥ 50 mm), the observed 5-year survival with preoperative stenting was 36.9%, compared to 39.6% without stenting (*p* = 0.561). The adjusted HR was 1.20 (95% CI 0.86–1.69) (Table 3).

### Secondary Outcomes

The observed 90-day mortality rate was 9.7% in patients with preoperative stent and 5.1% in patients without stent (Fig. 1). The adjusted HR of 90-day mortality was 2.49 (95% CI 1.27–4.87) in model 1 and 2.49 (95% CI 1.25–4.99) in model 2 (Table 2).

In subgroup analyses, the 90-day mortality rate in patients with locally advanced disease with and without preoperative stent was 8.7% and 4.0%, respectively. In adjusted analysis, preoperative stent was associated with a higher mortality hazard (HR 3.88, 95% CI 1.70–8.87) (Table 4).

**Table 4:** Hazard ratios (HRs) with 95% confidence intervals (CIs) of 90-day mortality comparing patients with esophageal cancer with and without preoperative stent in Finland in 1999–2016. Four subgroup analyses were performed

	Number of patients	With preoperative stent, HR (95% CI)	No preoperative stent, HR (95% CI)
Locally advanced disease (T3 and/or N +)			
90-day mortality			
All patients (crude)	696	2.22 (1.06–4.66)	1.00 (reference)
All patients (adjusted)*	696	3.88 (1.70–8.87)	1.00 (reference)
Neoadjuvant-treated patients			
90-day mortality			
All patients (crude)	502	3.41 (1.39–8.40)	1.00 (reference)
All patients (adjusted)*	502	3.99 (1.51–10.50)	1.00 (reference)
Clinical T3–4 tumors			
90-day mortality			
All patients (crude)	643	2.12 (1.04–4.32)	1.00 (reference)
All patients (adjusted)*	643	4.22 (1.85–9.62)	1.00 (reference)
Large tumors (≥ 50 mm)			
90-day mortality			
All patients (crude)	442	2.12 (0.83–5.39)	1.00 (reference)
All patients (adjusted)*	442	2.59 (0.95–7.06)	1.00 (reference)

^*^Adjusted for age (continuous), sex, year of the surgery (continuous), Charlson comorbidity score (0, 1, or ≥ 2), histology, pathological stage (0–I, II, III, or IV), and neoadjuvant therapy (yes, or no)

When only neoadjuvant-treated patients were included, those with and without preoperative stent had a 90-day mortality rate of 8.5% and 2.5%, respectively. In adjusted analysis, a higher mortality hazard was seen in stented patients (HR 3.99, 95% CI 1.51–10.50) (Table 4).

In the subgroup of patients with clinical T3–4 tumors, the 90-day mortality was 9.5% after preoperative stent and 4.6% without stent. In adjusted analysis, a higher mortality hazard was seen after preoperative stent (HR 4.22, 95% CI 1.85–9.62) (Table 4).

In the subgroup of large tumors (≥ 50 mm), the observed 90-day mortality rate was 8.6% after preoperative stent and 4.0% without stent. The adjusted HR was 2.59 (95% CI 0.95–7.06) (Table 4).

### Post hoc Analyses

To further explore the reasons behind the observed higher 90-day mortality after stenting, the highest percentiles of patients with preoperative stent and without stent were compared to those of operative time and bleeding. This post hoc analysis included only patients who received neoadjuvant treatment and was performed in order to resolve whether major intraoperative difficulties in some proportion of patients could explain mortality differences. The 50^th^, 75^th^, and 90^th^ percentile for bleeding in patients with preoperative stent was 424 ml, 700 ml, and 1200 ml, respectively. In patients without stent, respective bleeding amounts were 400 ml, 900 ml, and 1500 ml. Similarly, the 50^th^, 75^th^, and 90^th^ percentile in operation time in the stent group was 360 min, 423 min, and 512 min, respectively. Respective times in the no-stent group were 345 min, 398 min, and 455 min.

## Discussion

The present study suggests that patients who received preoperative esophageal stent had decreased 5-year overall survival after esophagectomy, after adjusting with confounding factors. Stenting was significantly associated with increased 90-day mortality, as well. It remains speculative whether increased postoperative mortality could be the result of stent itself, or if it could be explained with patient-related factors. Furthermore, worse 5-year overall survival in stented patients can be due to other risk factors and increased postoperative morbidity. This study cannot, however, exclude the possibility that stent can cause other adverse events, such as tumor cell seeding and metastatic behavior.

The main strength of this study is the population-based nationwide design with 100% follow-up information from the national registries. Confounding was taken into account by adjusting for key factors, including malnutrition based on albumin level, not usually available in registry studies, and BMI. Both the main analysis and multiple subgroup analyses showed significantly worse 5-year survival in the stent group. Still residual confounding is possible, since patient-related factors such as physical fitness^22^ and muscle loss^23^ could not be adjusted for. Also, although BMI was adjusted, a more important factor of the decrease in BMI could not be adjusted. Furthermore, despite adjusting for several confounders and subgroup analyses including tumor size, it is possible that obstructing tumors present more advanced disease independent of stenting. Even with 18-year nationwide data and a much higher number of patients with stents compared to previous studies assessing long-term outcomes, the sample size was barely sufficient and confidence intervals still include clinically significant point estimates. Additional studies, especially large randomized trials, would still provide valuable information.

Only one population-based study analyzing mid/long-term survival after stenting before esophagectomy exists.^14^ Regardless of a large background population of 2944 esophageal cancer patients, the final cohort, after propensity score matching with 1:4 ratio, included only 38 patients with stent and 152 without stent. They reported worse 3-year survival in the stent group, 25% vs. 44%, and higher 3-year locoregional recurrence rate, 62% vs. 34%.^14^ The reason behind worse long-term outcomes remains somewhat speculative and could reflect, for example, larger tumor size. R0 resection rate was significantly worse in the stent group, 71% vs. 86%, possibly explaining the majority of the observed long-term differences. Regardless of the propensity matching, the lower R0 resection rate in the stent group could reflect more advanced tumor growth. In our study with 134 stented patients, the R0 rate in stent and no-stent groups was 86.6% and 89.1%, respectively. The observed difference was small. It is reasonable, however, to conclude that this 2.5% lower R0 rate could partly explain the observed small survival differences in this study, which vary depending on selected analysis between 2.6 and 11.0% percentage points. On the other hand, in large tumors, no survival difference between stent and no-stent groups was seen. Stenting could, however, decrease survival also by causing microscopic tumor cell dissemination or even macroscopic tumor spread caused by esophageal perforation, although usually covered and controlled with the stent.^13^ In our series, macroscopic perforation occurred in 9 patients (6.7%) causing urgent operation. One small single-center 1:1 propensity-matched study with 30 patients per group showed no significant difference in overall survival between stent (28.5 months) and control (34 months) groups, but longer operative time was seen in stented patients (436 min vs. 375 min).^24^ As stent is associated with increased short-term mortality, it seems likely that postoperative morbidity and mortality affect also long-term results, also by reducing the number of patients receiving adjuvant treatment. Whether the increased short-term mortality is actually caused by stent, or is only an association to patient-related factors, remains to be answered. According to our data, however, stent was related to higher operation time, suggesting some intraoperative difficulties.

To date, the largest study to examine short-term outcomes in esophageal cancer patients with preoperative stent is a national Finnish and Swedish registry-based study.^15^ This study included locally advanced esophageal cancers from 2007 to 2014 with (127 patients) and without (902 patients) stent.^15^ Ninety-day mortality was higher without statistical significance in patients with preoperative stent (11.8% vs. 7.0%). In that study, the data of neoadjuvant therapy and many patient-related factors were lacking. A Danish single-center study, in which none of 273 locally advanced esophageal cancer patients received neoadjuvant therapy, reported no difference in postoperative complications or 30-day mortality rates in patients with (1/63, 1.6%) and without (5/210, 2.4%) stent.^25^ The previously mentioned French study^14^ with 60% of included patient having neoadjuvant therapy reported a higher number of Clavien-Dindo ≥ 3a complications (45% vs. 27%) and a non-significant trend towards increased in-hospital mortality (13.2% vs. 8.6%) in stented patients.^14^ In the current study with 76% of stented patients having neoadjuvant therapy, the observed 90-day mortality rate in patients with and without stent was 9.7% and 5.1%, respectively, and adjusted HRs were more than twofold with stent in most analyses. In neoadjuvant-treated patients, HR was fourfold with a stent compared to that of patients without stent. Therefore, based on this study and those previous large studies, it seems evident that, especially in patients undergoing neoadjuvant therapy, stenting is associated with an increased rate of postoperative mortality.

Several factors may explain the observed higher short-term mortality in stented patients. First, these patients have had severe dysphagia often with significant malnutrition. Stenting does not, however, seem to always improve nutritional status, measured by albumin and weight, associated with morbidity and mortality.^26^ Furthermore, in addition to major differences in albumin levels, BMI was as much as 4 units lower in the stent group in our study. Especially in stented patients, nutritional prehabilitation, which has been shown to improve perioperative functional capacity,^27^ could be important. A previous Japanese study showed higher mortality risk in both underweight and obese patients.^20^ We were able to adjust for this, but even higher risk is associated with weight and skeletal muscle loss,^23,28^ which could not be adjusted for, and therefore, included parameters do not represent all adverse aspects caused by malnutrition. Second, stents can cause inflammation, scar formation, and loss of normal anatomical planes complicating surgery. This theory of more difficult surgery after stenting is supported by observed longer operation time in patients with preoperative stent. Third, stents are often associated with severe reflux causing mucosal damage even high in the esophagus potentially risking the anastomotic healing. No data of leak rates after stenting was, however, available. Fourth, stenting can be related to a higher proportion of neck anastomoses, which are related to higher complication rates.^29^ This was not supported by our data, since no excess rate of neck anastomoses was seen in stented patients. Fifth, metallic stents produce high radial force.^30^ This might cause stent penetration to the mediastinum or to the aortic or airway wall during neoadjuvant therapy and eventually lead to serious adverse events during surgery.^13^ These sorts of major difficulties were suggested also by the nearly 1-h-longer operation time in the 90^th^ percentile in the stent group. Sixth, in our series, stent insertion–related perforations occurred in 9 patients, resulting with abandonment of neoadjuvant treatment and more urgent operation clearly affecting both short- and long-term results. Further studies assessing both intraoperative and postoperative complications related to stenting are needed.

European Society for Medical Oncology (ESMO) guideline^2^ takes strong opinion with level II evidence on stenting and cites a single propensity-matched study: “Endoscopic stenting should not be used in locoregional disease in operable patient and alternative routes of feeding (e.g. with needle catheter jejunostomy) should be preferred.”^14^ Though in the current study both short- and long-term results are worse after stenting, this not necessarily mean that we should avoid preoperative stenting. Especially since major risk factors, such as physical fitness, could not be adjusted for, it is possible that an excess 90-day mortality rate could be due to patient-related rather than stent-related factors. If so, preoperative stent could be an association rather than the cause. Alternatives for stenting are needle jejunostomy (proposed by ESMO guideline), nasogastric/nasojejunal feeding tubes, and gastrostomy. Since preoperative nutrition plays major role also in recovery, a reliable enteral nutrition route is needed during neoadjuvant treatment.^4^ Though the feasibility of feeding jejunostomy and gastrostomy has been reported with good success,^10,11^ certainty, according to a recent review, on the optimal feeding route during neoadjuvant treatment is lacking.^9^ In the recent retrospective study comparing stent and feeding jejunostomy, patients with jejunostomy returned to baseline weight faster, but both groups experienced high complication rates.^31^ In our series, 6.7% of stented patients suffered a perforation but this number needs to be compared with complications rates of jejunostomy. A direct comparison between different nutritional strategies in randomized controlled setting is needed before one feeding route can be declared superior to another. Esophageal stenting still cannot be ruled out with available knowledge.

## Conclusion

This population-based nationwide study from Finland reports decreased 5-year survival in patients with preoperative esophageal stent compared to those without stent. Stented patients also carry higher risk for 90-day mortality. It remains, however, possible that stent-related worse outcome could be only an association rather than the cause.
